# Case Report: A rare case of small bowel obstruction secondary to plasma cell myeloma

**DOI:** 10.3389/fonc.2022.934566

**Published:** 2022-08-05

**Authors:** Arturo Bonometti, Nicola Aronico, Giovanni Santacroce, Sara Fraticelli, Marco Lucioni, Claudio Salvatore Cartia, Alessandro Vanoli, Mario Andrea Latorre, Luca Arcaini, Marco Paulli, Antonio Di Sabatino

**Affiliations:** ^1^ Unit of Anatomic Pathology, Fondazione Istituto di Ricovero e Cura a Carattere Scientifico (IRCCS) Policlinico San Matteo, University of Pavia, Pavia, Italy; ^2^ Department of Molecular Medicine, University of Pavia, Pavia, Italy; ^3^ Pathology Unit, Humanitas Clinical and Research Center Istituto di Ricovero e Cura a Carattere Scientifico (IRCCS), Rozzano, Italy; ^4^ First Department of Internal Medicine, Fondazione Istituto di Ricovero e Cura a Carattere Scientifico (IRCCS) Policlinico San Matteo, University of Pavia, Pavia, Italy; ^5^ Division of Hematology, Fondazione Istituto di Ricovero e Cura a Carattere Scientifico (IRCCS) Policlinico San Matteo, Pavia, Italy

**Keywords:** chemotherapy, intestinal occlusion, plasma cell myeloma, small bowel, extramedullary presentation

## Abstract

Gastrointestinal (GI) involvement of plasma cell neoplasms is extremely rare. Herein, we describe the case of a 74-year-old Caucasian woman who came to our attention with abdominal pain, food vomiting, and weight loss of 10 kg over 1 year. A computed tomography scan of the abdomen revealed circumferential thickening of terminal ileum, for which the patient underwent an urgent 20-cm-long ileal resection. Histopathological and immunophenotypic analysis revealed a plasma cell neoplasm of the ileum. Subsequent investigations found a serum monoclonal immunoglobulin A component, an osteolytic lesion of the left jaw, and a clonal bone marrow plasma cell infiltrate carrying 1q21 amplification. Given the final diagnosis of plasma cell myeloma (PCM), the patient underwent a VMD (bortezomib, melphalan, and dexamethasone) chemotherapy regimen, achieving a complete remission after a 12-month treatment. For disease relapse, two further chemotherapy regimens were later attempted. At the last follow-up 4 years after the diagnosis, the patient is still alive. This case draws attention to the extramedullary presentation of plasma cell neoplasms, even if rare, as a prompt diagnosis seems to result in a better prognosis. In addition, it highlights the relevance of a multidisciplinary approach, involving gastroenterologists, hematologists, and pathologists, to the diagnosis and management of these neoplasms.

## Introduction

Plasma cell neoplasms are a clinically heterogeneous group of clonal disorders of terminally differentiated B cells, often characterized by the secretion of monoclonal immunoglobulins (Ig), and include plasma cell myeloma (PCM), solitary plasmacytoma of bone, and extraosseous plasmacytoma (1).

PCM is the plasma cell neoplasm with the highest clinical burden and possibly the worst outcome, and its diagnosis is based on the detection of more than 10% of clonal bone marrow plasma cells or biopsy-proven bony or extramedullary plasmacytoma, and any one or more of the myeloma defining events, namely, CRAB (hypercalcemia, renal insufficiency, anemia, and bone lesions) features and biomarkers of malignancy (specifically more than 60% clonal plasma cells on bone marrow examination, serum involved/uninvolved free light chain ratio of 100 or greater, and more than one focal lesion on magnetic resonance) (2).

The bone is typically involved in PCM, but other organs may be infiltrated as well (1). Gastrointestinal (GI) involvement of plasma cell neoplasms seems infrequent, generally as a part of a multisystemic involvement (3), and it is described by few reports and a single large series (4–8). The liver seems to be the organ of the GI tract most affected, whereas stomach or intestine is far less reported as disease sites. Interestingly, GI involvement appears to be associated with adverse biological features (e.g., high lactate dehydrogenase levels, high risk karyotype, and plasmablastic cytology) and an aggressive behavior (4, 9).

Herein, we report a case of PCM presenting with ileal obstruction and diagnosed following emergency surgery.

## Case presentation

A 74-year-old Caucasian woman referred to our centre in September 2017 for abdominal pain, food vomiting, and weight loss of 10 kg over 1 year. She had a medical history of arterial hypertension, hypercholesterolemia, obesity (with a body mass index of 31.2 and a body surface area of 1.89), primary osteoporosis, chronic Helicobacter pylori-unrelated gastritis, and smoking habit. Medications taken at home were antihypertensive drugs, hypolipidemic agents, and proton pump inhibitors. For osteoporosis, the patient was on calcium and vitamin D supplementation therapy. A previous exposure to radiation and chemical agents was excluded.

**Figure 1:**
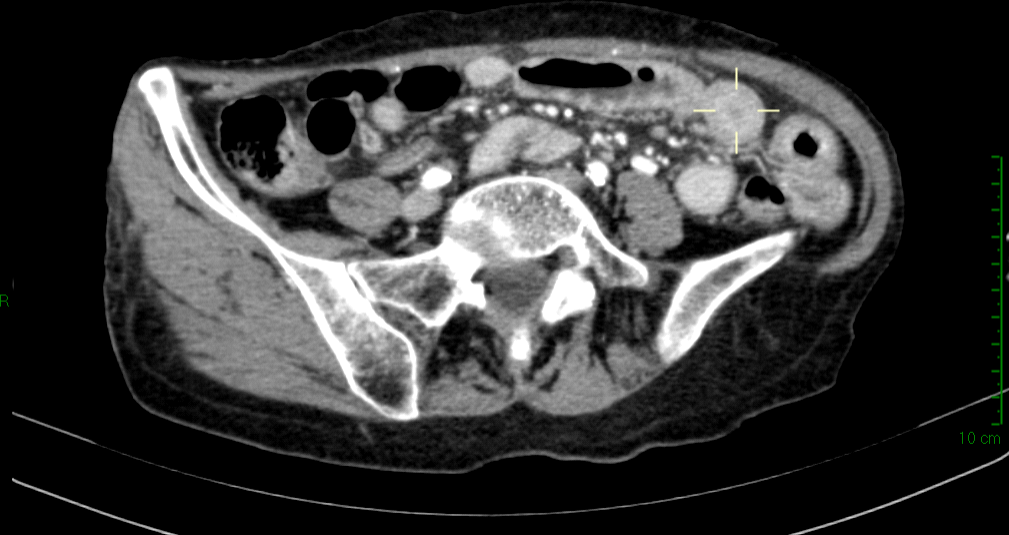
Abdominal computed tomography scan showing wall circumferential thickening (delimited by the pointer) at terminal ileum.

A computed tomography (CT) scan of the abdomen showed a small bowel circumferential wall thickening (**Figure 1**), for which the patient underwent an urgent ileal resection by laparoscopy. The surgical specimen consisted of a 20-cm-long segment of terminal ileum. At gross examination, the ileal wall displayed a 3-cm-long ulcerated lesion, determining a stenosis of the bowel lumen and infiltrating the mesentery through the visceral wall.

**Figure 2:**
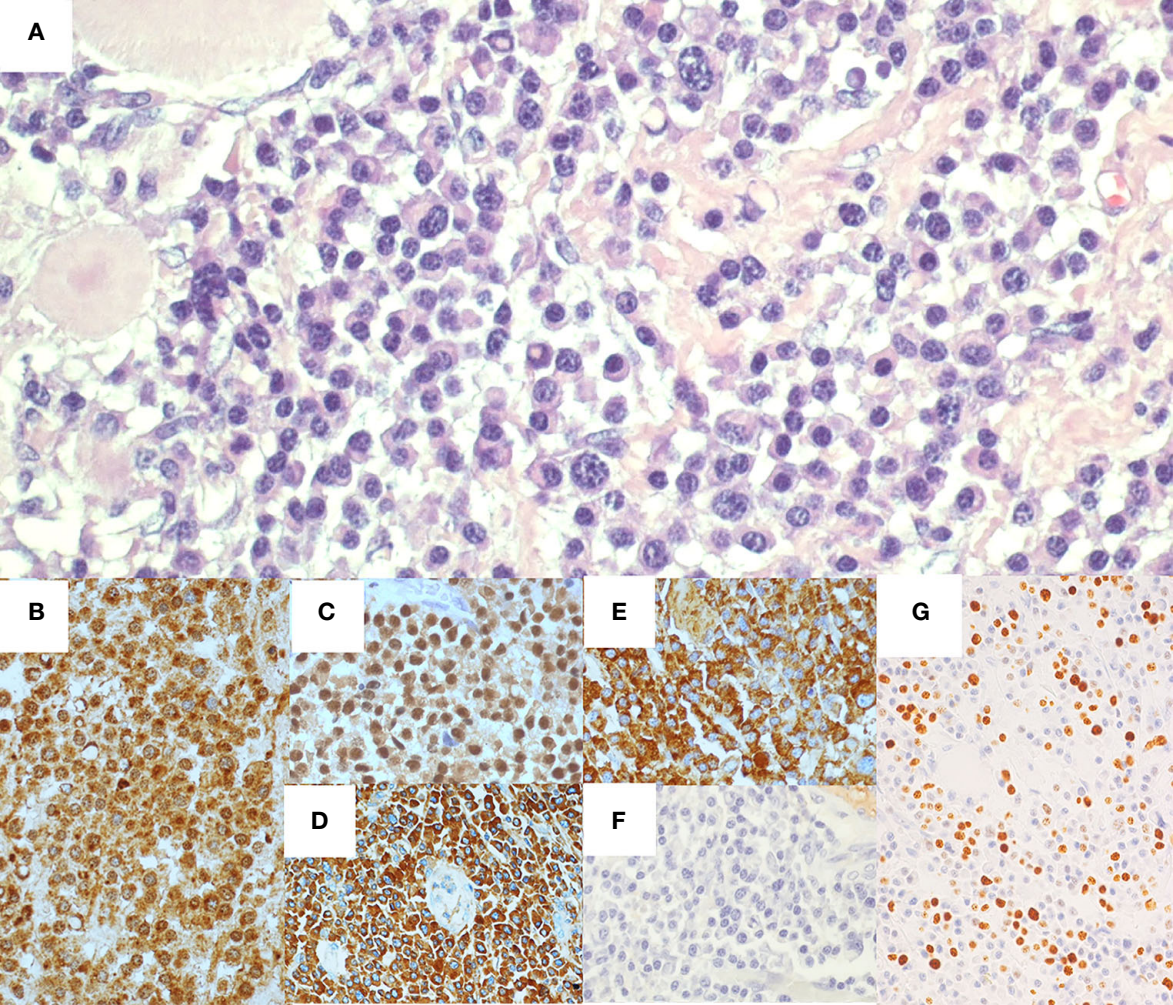
Histological and immunohistochemical features of ileal resection specimen. Hematoxylin-eosin staining **(A)** shows ileal wall infiltrated by a proliferation of pleomorphic plasma cells. Immunohistochemical staining presented CD138^+^ **(B)**, MUM1^+^ **(C)**, IgA^+^ **(D)**, and monoclonal restriction for Kappa light chain of Ig **(E)**, whereas Lambda light chains of Ig tested negative **(F)**. The proliferative index (Mib1/Ki67) was around 40% **(G)**.

Microscopic analysis of the specimen (**Figure 2**) revealed a dense trans-parietal pleomorphic proliferation of mature plasma cells, consisting of small to large and multinucleated elements, some with Russel or Dutcher bodies, with ulceration of the bowel mucosa and infiltration of the perivisceral adipose tissue. All those elements stained positive for CD56, CD79a, CD138, MUM1, IgA and showed monoclonal restriction for the kappa (κ) light chain of Ig. CD5, CD20, CD45/LCA, Cyclin-D1, IgD, IgG, and IgM tested all negative as well as immunostaining for human herpes virus 8 (HHV8) and *in situ* hybridization for Epstein–Barr virus (EBV). The proliferative index of the neoplastic population (Mib1/Ki67) was estimated at around 40%. A moderate number of CD3^+^ T lymphocytes were admixed to the plasma cell infiltrate. All the resected lymph nodes were spared, displaying reactive features.

**Figure 3:**
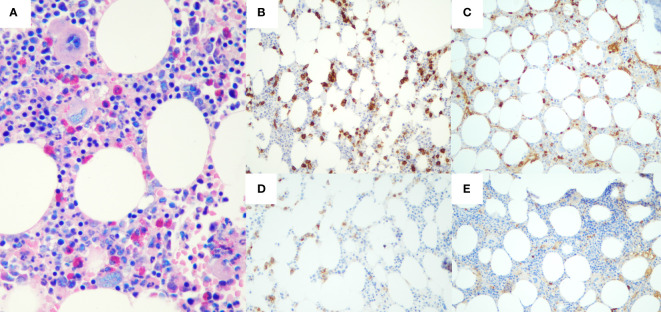
Histological and immunohistochemical features of bone marrow biopsy. Hematoxylin-eosin staining **(A)** shows a plasma cell infiltrate, accounting for 15% of bone marrow cellularity. Immunohistochemical staining presented CD138^+^ **(B)**, with restriction for Kappa light chain of Ig **(C)**. Neoplastic plasma cells also aberrantly expressed CD56 **(D)**, whereas immunohistochemistry for Lambda light chain resulted completely negative **(E)**.

Laboratory investigations were all within the limits (**Table 1**), with the exception of the serological presence of a monoclonal IgA-κ component equal to 1.2 g/dl with uninvolved normal Ig (IgG, 790 mg/dl; IgA, 1,380 mg/dl; IgM, 250 mg/dl) and antinuclear antibody positivity. Moreover, microscopic analysis of the bone marrow biopsy (**Figure 3**) showed a clonal plasma cell infiltrate (15% of bone marrow cellularity), consisting of small- to medium-sized plasma cells with aberrant expression of CD56 and monoclonal restriction for κ light chain of Ig, without amyloid deposits. Fluorescence *in situ* hybridization analysis documented the presence of 1q21 amplification.

**Table 1:** Timeline of the disease and treatment.

Timeline	Diagnosis and Treatment	Outcome
**September 2017**	- *CT scan:* small bowel circumferential wall thickening - *Laparoscopic ileal resection:* proliferation of mature plasma cells	
**November 2017**	- *Bone marrow biopsy:* bone marrow involvement by plasma cell neoplasm (15%) - *Laboratory exams:* monoclonal IgA-κ component, 1.2 g/dl; TP, 7.6 g/dl; alb, 4.6 g/dl; γ−globulins, 1.7 g/dl; CBC (Hb, 13 g/dl; WBC, 7060/mmc; NEU, 3880/mmc; PLT, 221000/mmc); LDH, 144 UI/L; Beta-2 microglobulin, 3.7 mg/L; creatinine, 0.9 mg/dl; calcemia, 10.4 mg/dl; IFU + κ chain; normal urine exam; FLC κ, 239 mg/L; FLC lambda, 15.4 mg/L; ratio, 15.51; IgG, 790 mg/dl; IgA, 1,380 mg/dl; IgM, 250 mg/dl - *FISH analysis:* presence of 1q21 amplification - *Total body CT scan:* osteolytic lesion of the left jaw (confirmed on positron emission tomography)	Final diagnosis: plasma cell myeloma with ileal involvement
**January 2018 to November 2018**	- Chemotherapy with bortezomib, melphalan and dexamethasone (VMD for nine cycles)	Complete response based on IMWG criteria
**May 2020**	- *Serological and skeletal relapse:* multiple bone involvement with pathological vertebral fractures and presence of monoclonal IgA-κ component - *Bone marrow biopsy:* bone marrow involvement by plasma cell neoplasm (30%)	
**July 2020 to July 2021**	- Second-line chemotherapy with daratumumab, lenalidomide, and dexamethasone(Dara-Rd for14 cycles)	Progressive disease based on IMWG criteria (only serological)
**September 2021 to ongoing**	- Third-line chemotherapy with pomalidomide, bortezomib, and dexamethasone (PVD 10 cycles: ongoing)	Alive at last FU (April 2022)

CT, computed tomography; Ig, immunoglobulins; κ, kappa; TP, total proteins; alb, albumin; CBC, cell blood count; Hb, hemoglobin; WBC, leukocytes; NEU, neutrophils; PLT, platelet; IFU, immunofixation of urine; FLC, free light chain; FISH, fluorescence in situ hybridization; IMWG, International Myeloma Working Group; FU, follow-up.

A total body CT showed an osteolytic lesion of the left jaw, confirmed on positron emission tomography (PET) scan as secondary to the disease process. An upper GI endoscopy showing no pathological findings completed the diagnostic workup.

Our final diagnosis was PCM with ileal involvement. Therefore, the patient received chemotherapy according to the VMD scheme (i.e., bortezomib, 1.3 mg/m^2^; melphalan, 0.12 mg/kg; and dexamethasone, 20 mg for nine cycles), achieving a complete remission in a 12-month period according to the International Myeloma Working Group consensus criteria (10). Thirty months later, a disease relapse was highlighted by the findings of increased monoclonal IgA-κ component, multiple lytic vertebral lesions showed by magnetic resonance imaging (MRI), and bone marrow plasma cell infiltration equal to 30%. Then, the patient underwent a second-line chemotherapy with Dara-Rd scheme (i.e., daratumumab, 16 mg/kg; lenalidomide, 5 mg per day; and dexamethasone, 20 mg for week) for 14 cycles achieving a partial remission. A third-line therapy was started on September 2021 due to only serological progressive disease without new lytic lesions based on PVD scheme (i.e., pomalidomide, 4 mg per day; bortezomib, 1.3 mg/m^2^; and dexamethasone, 40 mg per week). At the last follow-up, 4 years after the diagnosis, the patient received the 10th cycle of PVD reaching at least a partial response.

The clinical course of the patient is summarized in **Table 1**.

## Discussion

Plasma cell neoplasms are categorized into four groups: PCM (including smoldering and non-secretory myeloma and plasma cell leukemia) and plasmacytoma [including solitary plasmacytoma of bone and extraosseous plasmacytoma (EP)] (1). EP is rare representing only 5% of all plasma cell neoplasms and generally involves mucous membranes of airways or GI tract and only rarely parenchimatous organs (11, 12). It is more common in men, with a male-to-female ratio of 4:1 with a median patient age of 55 years at diagnosis (11, 12). The diagnosis of EP needs to exclude a systemic involvement by a PCM, and it is therefore a diagnosis of exclusion. Therefore, EP needs to be distinguished by extraosseous involvement by a PCM (1). In such case, the most frequently involved tissues are soft tissues, skin, lymph nodes, liver, spleen, and kidneys. Conversely, GI involvement by a PCM is very rare. It most commonly occurs in the small intestine, followed by stomach, whereas the involvement of esophagus and colon—especially in ileo-cecal region—is exceedingly rare (1, 4, 6, 7, 9). In the literature, very few cases of multiple myeloma primarily presented with GI involvement are reported (**Table 2**). GI involvement by PCM may occur with loss of appetite, bleeding, abdominal discomfort, or obstruction (4, 13, 14). In such cases, histopathological examination of tissue samples is needed to address the diagnosis of plasma cell neoplasms, whereas imaging (CT scan, MRI, and PET-CT) together with laboratory examination and bone marrow biopsy may confirm the presence of a systemic involvement (4).

**Table 2:** Case reports of PCM with gastrointestinal tract involvement at presentation.

Pt	Sex	Age (Years)	GI Site	Clinical Presentation	BMPC Infiltration	Chromosomal Abnormalities	Therapy	Outcome(Time)	Ref
1	F	74	Ileum	Obstruction	15%	1q21	VMD, Dara-Rd, PVD	Alive (4 years)	Present case
2	n.a.	n.a.	Stomach and Rectum	Upper GI bleeding	n.a.	n.a.	n.a.	n.a.	4
3	n.a.	n.a.	Right Colon	Weight loss and Abdominal discomfort	n.a.	Complex	n.a.	n.a.	4
4	n.a.	n.a.	Rectum	Tenesmus and Hematochezia	n.a.	n.a.	n.a.	n.a.	4
5	M	79	Colon	Obstruction	n.a.	n.a.	Surgery	Dead (2 months)	13
6	M	68	Stomach	Upper GI bleeding	n.a.	n.a.	Refused	Dead (1 month)	14

BMPC, bone marrow plasma cell; F, female; GI, gastrointestinal; M, male; n.a., not available; PCM, plasma cell myeloma; Pt, patient; Ref, reference; Dara-Rd, daratumumab; lenalidomide and dexamethasone scheme; PVD, pomalidomide, bortezomib and dexamethasone scheme; VMD, bortezomib, melphalan, and dexamethasone scheme.

This is indeed the case of our patient, in which the ileal involvement by a plasma cell neoplasm emerged after the development of GI symptoms such as abdominal pain, food vomiting, and weight loss of 10 kg over 1 year. The diagnosis of a GI involvement by PCM can be difficult from both a clinical and histopathological point of view, as it can mimic a carcinoma or lymphoma.

Our first diagnostic hypothesis, based on the patient’s age, clinical presentation, and CT finding of GI obstruction, was intestinal neoplasm. Given the ileal localization, a neuroendocrine tumor, an intestinal lymphoma, or an adenocarcinoma was highly suspected. The subsequent urgent ileal resection and histological examination led to the more unusual diagnosis of plasma cell neoplasia.

This case required a histopathological complex differential diagnosis between plasma cell neoplasms and lymphomas with predominant plasma cell differentiation and possible GI involvement, namely, marginal zone lymphoma and several high-grade B-cell lymphoma (i.e., plasmablastic lymphoma, EBV^+^ diffuse large B-cell lymphoma, and HHV8^+^ B-cell lymphoma) (1). In our patient, the morphologic and immunohistochemical features of the neoplastic infiltrate, the absence of large cells with blastic appearance, and the negativity for both EBV and HHV8 search suggested a PCM rather than the aforementioned entities. The integration with clinical features was necessary to conclude the differential diagnosis. In particular, the absence of history of immunosuppression ruled out the plasmablastic lymphoma, whereas the serological presence of a monoclonal IgA-κ component, the imaging detection of osteolytic lesions, and the bone marrow plasma cell infiltration allowed the final diagnosis of PCM.

From a clinical perspective, a differential diagnosis between PCM and extraosseous plasmacytoma was necessary. As mentioned above, serological and imaging examinations, associated with the bone marrow biopsy, showed the neoplasm systemic involvement in our patient, allowing the diagnosis of PCM and the exclusion of extraosseous plasmacytoma.

According to the available data, PCM with intestinal involvement has a poor prognosis, with a median survival of only a few months, despite the recent discovery of novel active agents (4, 9). In addition, the chromosome 1 abnormalities, such as the 1q21 amplification of our patient, frequently detected in PCM with extramedullary involvement, are typically associated with a worse prognosis (15, 16). Our patient, despite the presence of such negative prognostic factors, is still alive at the last follow-up 4 years after diagnosis. We assume that early diagnosis of PCM by GI presentation, with limited bone co-involvement, and subsequent prompt targeted chemotherapy, resulted in a significant prognostic improvement.

In conclusion, although intestinal involvement of PCM is rare, it must be promptly identified, and a multidisciplinary approach, involving gastroenterologists, hematologists, and pathologists, is required to achieve a proper diagnosis and a successful management. In addition, this report highlights that an appropriate and early diagnosis is associated with a prognostic improvement, although further studies on a large number of patients are needed to better clarify this issue.

## Data availability statement

The original contributions presented in the study are included in the article/supplementary material. Further inquiries can be directed to the corresponding author.

## Ethics statement

The studies involving human participants were reviewed and approved by San Matteo Hospital Foundation Pavia Ethics Committee. The patients/participants provided their written informed consent to participate in this study. Written informed consent was obtained from the individual(s) for the publication of any potentially identifiable images or data included in this article.

## Author contributions

AB, NA, and ML designed and drafted the report. AB, GS, AV, CC, SF, MAL, LA, and MP collected, analyzed, and interpreted the data; AS and MP critically revised the manuscript. All authors contributed to the article and approved the submitted version.

## Funding

This project was funded by “San Matteo Hospital Foundation, Internal Medicine research fundings”.

## Conflict of interest

The authors declare that the research was conducted in the absence of any commercial or financial relationships that could be construed as a potential conflict of interest.

## Publisher’s note

All claims expressed in this article are solely those of the authors and do not necessarily represent those of their affiliated organizations, or those of the publisher, the editors and the reviewers. Any product that may be evaluated in this article, or claim that may be made by its manufacturer, is not guaranteed or endorsed by the publisher.
